# Maternal Engagement in HIV Care and Antiretroviral Therapy Uptake Among Children Living With HIV

**DOI:** 10.1001/jamanetworkopen.2026.30983

**Published:** 2026-08-26

**Authors:** Jiawei He, Bricia Gonzalez Trejo, Kemal Sherefa Oumer, Amanda Movo, Simon I. Hay, Christopher J. L. Murray, Peng Zheng, Aleksandr Y. Aravkin, Jeffrey W. Imai-Eaton, Hmwe H. Kyu

**Affiliations:** 1 Institute for Health Metrics and Evaluation, University of Washington, Seattle; 2 Department of Health Metrics Sciences, School of Medicine, University of Washington, Seattle; 3 Center for Communicable Disease Dynamics, Department of Epidemiology, Harvard T.H. Chan School of Public Health, Boston, Massachusetts; 4 MRC Centre for Global Infectious Disease Analysis, School of Public Health, Imperial College London, London, United Kingdom

## Abstract

**Importance:**

Children living with HIV (CLHIV) in sub-Saharan Africa have lower rates of antiretroviral therapy (ART) uptake and viral load suppression (VLS) than adults with HIV. The association of maternal engagement in HIV care with child VLS remains underexplored.

**Objective:**

To estimate the prevalence of ART uptake and VLS among CLHIV, and to examine the association between maternal engagement in HIV care and key steps in the pediatric HIV care cascade.

**Design, Setting, and Participants:**

This cross-sectional study was conducted from December 2025 to February 2026 using data from the nationally representative, cross-sectional, household-based Population-Based HIV Impact Assessment (PHIA) surveys administered in 13 countries in sub-Saharan Africa between 2015 and 2019. Participants were selected using a multistage stratified cluster sampling design and were restricted to mother-child dyads including CLHIV younger than 15 years with known ART and VLS status.

**Exposure:**

Maternal engagement in HIV care, defined as maternal ART uptake and VLS (assessed via participant self-report and laboratory blood specimen analysis).

**Main Outcomes and Measures:**

Country-specific prevalence of ART and VLS among CLHIV and the association between maternal care engagement and child VLS. Post hoc hypotheses were tested using logistic regression, which was adjusted for complex survey design and confounders.

**Results:**

Across the 13 surveys, 688 CLHIV (353 females [51.3%]; mean [SD] age, 8.3 [4.0] years) had data on ART uptake and VLS status and had linkable maternal data. Two mother-child dyads were excluded due to missing maternal survival data, resulting in a final sample of 686. Pediatric ART uptake and VLS were lowest among children aged 0 to 4 years (69 [48.6%] and 39 [27.5%]) and children with young mothers aged 15 to 24 years (19 [54.3%] and 8 [22.9%]). VLS was significantly higher among children whose mothers had viral suppression compared with those whose mothers did not (185 [59.5%] vs 26 [24.1%]). ART uptake among CLHIV ranged from 83.5% (95% CI, 70.7%-91.4%) in Namibia to 46.0% (95% CI, 35.0%-57.5%) in Zambia in surveys sampling all children younger than 15 years. VLS prevalence ranged from a high of 75.4% (95% CI, 60.7%-85.9%) in Namibia to a low of 30.4% (95% CI, 12.5%-57.3%) in Tanzania. In multivariable analyses, maternal ART uptake was associated with child VLS (odds ratio [OR], 3.99; 95% CI, 1.63-9.78), but this association was attenuated after adjusting for child ART uptake (OR, 0.88; 95% CI, 0.30-2.60); findings were similar for maternal VLS. However, children whose mothers were receiving ART had significantly higher odds of using ART themselves (OR, 20.42; 95% CI, 8.77-47.54) as did children of mothers with VLS (OR, 5.29; 95% CI, 2.95-9.48).

**Conclusions and Relevance:**

This cross-sectional study found that maternal ART uptake and VLS were associated with VLS in CLHIV primarily through the child’s own ART uptake. Family-centered interventions integrating maternal and pediatric HIV care may support progress toward the UNAIDS 95-95-95 HIV targets.

## Key Points

**Question:**

Is maternal engagement in HIV care—specifically antiretroviral therapy (ART) and viral load suppression (VLS)—associated with VLS in children living with HIV?

**Findings:**

In this cross-sectional study of 686 mother-child dyads from 13 sub-Saharan African countries, mothers receiving ART and mothers with VLS were more likely to have children who use ART, which substantially increases the likelihood of child VLS.

**Meaning:**

The findings of this study suggest that maternal engagement in HIV care was associated with improved child VLS indirectly through increased child ART uptake, highlighting the critical role of integrated family-centered HIV services.

## Teaser

This cross-sectional study examines the role of antiretroviral therapy (ART) use and viral load suppression among mothers in their children’s viral load suppression in sub-Saharan Africa.

## Introduction

Despite progress over the past decade, pediatric HIV remains a major concern, particularly in sub-Saharan Africa. In 2024, 1.4 million children younger than 15 years lived with HIV globally.^1,2^ New infections have decreased by 58% since 2010,^3^ owing to the combination of declining HIV prevalence among pregnant women, successful scale-up of antenatal HIV testing, and the use of antiretrovirals to prevent vertical transmission during pregnancy and breastfeeding.^4^ However, more than 80% of new infections and AIDS-related deaths among children occur in sub-Saharan Africa,^5,6,7^ where only 56% of children younger than 15 years use antiretroviral therapy (ART) and even fewer are virally suppressed.^8^ Recent findings demonstrate a persistent gap in viral load suppression (VLS) among children in sub-Saharan Africa,^9^ highlighting the need to address pediatric HIV care within this region.

While the 2010 World Health Organization guidelines recommended immediate ART initiation for all children living with HIV (CLHIV), regardless of clinical or immunologic status,^10^ children remain less likely than adults to receive ART.^11,12,13,14,15^ In 2024, treatment coverage was 55% for children vs 78% for adults.^2^ Among those receiving treatment, children also have lower VLS rates.^16,17^ High virological failure and low VLS rates among children receiving ART have been documented in several countries.^17,18^ Early detection and management of nonsuppressed viral load are crucial, as virologic failure has been associated with rapid progression to AIDS and mortality.^19^ These persistent gaps in ART coverage and VLS among children underscore the need for targeted strategies to accelerate progress toward achieving global HIV targets.

Many studies on pediatric VLS in sub-Saharan Africa highlight age, ART treatment duration, CD4 count and viral load count at ART initiation, type of treatment, educational level, and sex as key factors.^9^ Parental factors studied have been limited to employment status, age, marital status, and educational level.^20,21,22,23,24^ Research linking maternal ART and VLS status to child HIV outcomes is scarce. One such study in western Kenya found children had twice the odds of nonsuppression if their caregivers did not have suppression.^25^ Another study in 7 sub-Saharan African countries found that 76% of HIV-positive mothers whose children had been diagnosed with HIV achieved VLS compared with only 45% of HIV-positive mothers with undiagnosed HIV-positive children.^26^ These findings suggest a potential association between maternal HIV care and child HIV outcomes and highlight the need for more comprehensive studies on the role of maternal engagement in care in shaping child HIV outcomes.

This study aimed to estimate the prevalence of ART uptake and VLS among CLHIV and to examine the associations between maternal engagement in HIV care and key steps in the pediatric HIV care cascade across 13 sub-Saharan African countries in the late 2010s. Quantifying the associations of maternal engagement factors with the HIV care cascade among CLHIV is critical for informing public health interventions aimed at reducing pediatric HIV-related illnesses and deaths.

## Methods

### Data Sources

Between December 2025 and February 2026, we conducted a secondary analysis of data from the Population-based HIV Impact Assessment (PHIA) surveys. The PHIA surveys received ethical approval from the institutional review boards and ethics committees at each participating sub-Saharan African nation and from the US Centers for Disease Control and Prevention.^27^ Informed consent from parents or guardians and assent from children were obtained by primary investigators during the original PHIA survey implementation. In accordance with 45 CFR §46.104, this cross-sectional study was exempt from ethics review and the informed consent requirement because the analysis used deidentified, publicly available data and did not constitute human participant research. We followed the Strengthening the Reporting of Observational Studies in Epidemiology (STROBE) reporting guideline^28^ and the Guidelines for Accurate and Transparent Health Estimates Reporting (GATHER).^29^

The PHIA surveys were nationally representative, cross-sectional, household-based surveys designed to measure HIV program impact and capture the state of the HIV epidemic among adults aged 15 to 64 years and children aged 0 to 14 years (with slight variations by survey). More information about the PHIA surveys is available elsewhere.^30^ Between 2015 and 2019, the PHIA surveys in 13 sub-Saharan African countries included HIV serologic and viral load testing among children. Households were randomly selected from primary sampling units within Census-derived enumeration units. Within all sampled households, all eligible adults aged 15 years or older were included, and either a subsample of eligible children younger than 15 years or all children in selected households were included. Additional details about the PHIA survey and differences in sampling of children by country are provided in eMethods 1 and eTable 1 in Supplement 1.

### Procedures

For our secondary analysis, we included survey data from Cameroon (2017-2018), Côte d’Ivoire (2017-2018), Eswatini (2016-2017), Ethiopia (2017-2018), Kenya (2018), Lesotho (2016-2017), Malawi (2015-2016), Namibia (2017), Rwanda (2018-2019), Tanzania (2016-2017), Uganda (2016-2017), Zambia (2016), and Zimbabwe (2015-2016). All surveys included a sample of children 0 to 14 years of age, except Rwanda, which included only children 10 to 14 years of age.

We included HIV-positive children with documented ART status (receiving ART or not receiving ART) and VLS status (suppressed or unsuppressed) who had linkable maternal data (regardless of maternal survival and survey participation). Maternal information was incompletely observed in the survey, limiting the sample size (eMethods 2 and eFigure 1 in Supplement 1). The distributions of ART uptake and VLS status among children who were included vs excluded in this study are available in eFigure 2 in Supplement 1. We further limited the analytic sample in the regression analysis by excluding 2 observations in which the mother’s survival status was unknown. ART status of children was ascertained from antiretroviral drug detection or caregiver report. Following World Health Organization guidelines, VLS was defined as less than 1000 copies/mL and unsuppressed viral load was at least 1000 copies/mL.^31^ Children with unknown ART status were classified as not receiving ART to calculate prevalence estimates and were otherwise excluded from this analysis. As an alternative analysis, we calculated the prevalence of ART uptake only in children with known treatment status (complete-case analysis). The main maternal factors of interest were maternal ART status (receiving ART, not receiving ART, mother alive but with unknown ART status, or mother deceased) and maternal VLS (suppressed, unsuppressed, mother alive but with unknown VLS status, or mother deceased). More details about how each variable was operationalized are provided in eMethods 3 in Supplement 1. Covariates included child sex (male vs female), child age category (0-4, 5-9, and 10-14 years), place of residence (rural vs urban), and maternal age category (15-24, 25-34, 35-49, and 50-64 years).

### Statistical Analysis

This analysis involved 3 components. In component 1, we described the subsample of children with linkable maternal data. We estimated the prevalence of ART uptake and VLS among all CLHIV by country. Prevalence of ART uptake was estimated among all HIV-positive children. Prevalence of child VLS was estimated among all HIV-positive children receiving ART.

In component 2, to assess whether maternal engagement in HIV care was associated with VLS among CLHIV after adjusting for covariates, we restricted the analytic sample of mother-child dyads to those cases in which the mother’s survival status was known (n = 686). Maternal ART status and maternal VLS were highly correlated (Pearson correlation coefficient = 0.997). To avoid multicollinearity and unstable estimates, we fit separate multivariable logistic regression models with maternal ART status as the primary exposure (model 1a) and maternal VLS as the primary exposure (model 2a) adjusted for prespecified covariates, including child sex, child age, place of residence, and maternal age. Model 1b and model 2b were further adjusted for child ART uptake. Estimates were reported as odds ratios (ORs) with 95% CIs. Statistical significance was defined as 2-sided *P* < .05. Detailed explanation of these models is provided in eMethods 4 in Supplement 1. In a sensitivity analysis, we calculated results for models in which the association between maternal factors and child VLS was restricted to children receiving ART, instead of adjusting for child ART status (eMethods 5 and eTable 2 in Supplement 1).

In component 3, using a conceptual framework linking maternal HIV care to the pediatric HIV care cascade (eFigure 3 in Supplement 1), we explored the hypothesized association between maternal engagement in HIV care and child VLS. Prior research from the prevention of mother-to-child transmission cascade found that maternal engagement in HIV care is associated with early infant diagnosis, a precursor for infant ART initiation and subsequent VLS.^32^ Building on these findings, we first assessed the association between maternal engagement in HIV care and child ART status (maternal ART uptake in model 3 and maternal VLS in model 4). For this analysis, we included the same 686 mother-child dyads in which the mother’s survival status was known. To complete the hypothesized pathway from maternal factors to pediatric outcomes, we validated the expected association between child ART status and VLS within our study population (eTable 3 in Supplement 1). As an alternate analysis, we performed a formal mediation analysis decomposing the total association of maternal engagement with child VLS into direct and indirect (through child ART uptake) components, using the product-of-coefficients (Sobel) method on the logit scale with the delta-method 95% CIs (eMethods 6 and eTable 4 in Supplement 1).^33,34^

All analyses were conducted in R, version 4.5.2 (R Project for Statistical Computing). All analyses accounted for the complex survey design that included clustering, stratification, and sampling weights. To account for the cross-national structure and ensure that country-level clustering was preserved beyond simple stratification, we nested all survey design elements by hierarchy within country. Primary sampling units were specified as the explicit interaction between country and sampling unit and stratification as the interaction between country and sampling stratum. Variance estimates were calculated using Taylor linearization to reflect the multistage sampling design. Descriptive statistics, including prevalence of key indicators, such as VLS, were estimated using the svyciprop function in the survey package, version 4.4.8 (R Project for Statistical Computing).^35^ Design-based 95% CIs were reported to quantify statistical uncertainty from the complex survey design. For regression analyses, we fitted multivariable logistic regression models using the svyglm function with quasibinomial family. Because these models are survey-weighted, ordinary likelihood–based Akaike information criterion (AIC), bayesian information criterion (BIC), and pseudo-*R*^2^ metrics were not defined; we therefore evaluated model performance and calibration using design-based AIC and BIC, the survey-weighted C statistic (area under the receiver operating characteristic curve), and the Archer-Lemeshow *F*-adjusted goodness-of-fit test. To evaluate potential multicollinearity among factors, we calculated Generalized Variance Inflation Factors adjusted for variable degrees of freedom (eTable 5 in Supplement 1). Normalized survey weights were applied throughout to account for differential country population sizes, differential sampling probabilities, and nonresponse. In a final sensitivity analysis, we restricted the sample in components 2 and 3 to mother-child dyads in which the mother was alive and lived in the same household (eMethods 7 and eTables 6 and 7 in Supplement 1).

## Results

### Component 1

Across the 13 PHIA surveys, 688 CLHIV (younger than 15 years) with data on ART and VLS status had linkable maternal data (Table 1). These children included 335 males (48.7%) and 353 females (51.3%), with a mean (SD) age of 8.3 (4.0) years. Two mother-child dyads were excluded due to missing maternal survival data, resulting in a final sample of 686.

**Table 1.** Unweighted Characteristics of Children Living With HIV and Their Mothers Participating in 13 PHIA Surveys, 2015-2019

Variable	CLHIV, No. (%)						
	Total No.	ART uptake			VLS^a^		
		Yes	No	*P* value	Yes	No	*P* value
Overall	688	486 (70.6)	202 (29.4)	NA	345 (50.1)	343 (49.9)	NA
**Child characteristics **							
Sex							
Male	335	246 (73.4)	89 (26.6)	.06	160 (47.8)	175 (52.2)	.12
Female	353	240 (68.0)	113 (32.0)		185 (52.4)	168 (47.6)	
Age group, y							
0-4	142	69 (48.6)	73 (51.4)	<.001	39 (27.5)	103 (72.5)	<.001
5-9	240	178 (74.2)	62 (25.8)		126 (52.5)	114 (47.5)	
10-14	306	239 (78.1)	67 (21.9)		180 (58.8)	126 (41.2)	
Place of residence							
Urban	195	131 (67.2)	64 (32.8)	.82	87 (44.6)	108 (55.4)	.045
Rural	493	355 (72.0)	138 (28.0)		258 (52.3)	235 (47.7)	
Wealth quintile							
First, lowest	115	89 (77.4)	26 (22.6)	.498	59 (51.3)	56 (48.7)	.11
Second	152	107 (70.4)	45 (29.6)		89 (58.6)	63 (41.4)	
Third	84	55 (65.5)	29 (34.5)		40 (47.6)	44 (52.4)	
Fourth	149	105 (70.5)	44 (29.5)		75 (50.3)	74 (49.7)	
Fifth, highest	188	130 (69.1)	58 (30.9)		82 (43.6)	106 (56.4)	
ART status							
Yes	486	NA	NA	<.001	325 (66.9)	161 (33.1)	<.001
No	202	NA	NA		20 (9.9)	182 (90.1)	
**Maternal characteristics**							
ART status							
No	63	11 (17.5)	52 (82.5)	<.001	10 (15.9)	53 (84.1)	<.001
Yes	357	294 (82.4)	63 (17.6)		202 (56.6)	155 (43.4)	
Alive but with unknown ART status	172	111 (64.5)	61 (35.5)		83 (48.3)	89 (51.7)	
Deceased	94	68 (72.3)	26 (27.7)		50 (53.2)	44 (46.8)	
Unknown survival	2	2 (100)	0 (0)		0 (0.0)	2 (100)	
VLS status							
No	108	47 (43.5)	61 (56.5)	<.001	26 (24.1)	82 (75.9)	<.001
Yes	311	258 (83.0)	53 (17.0)		185 (59.5)	126 (40.5)	
Alive but with unknown VLS status	173	111 (64.2)			84 (48.6)	89 (51.4)	
Deceased	94	68 (72.3)	26 (27.7)		50 (53.2)	44 (46.8)	
Unknown survival	2	2 (100)	0 (0)		0 (0)	2 (100.0)	
Age group, y							
15-24	35	19 (54.3)	16 (45.7)	.01	8 (22.9)	27 (77.1)	.04
25-34	289	185 (64.0)	104 (36.0)		135 (46.7)	154 (53.3)	
35-49	345	268 (77.7)	77 (22.3)		191 (55.4)	154 (44.6)	
50-64	19	14 (73.7)	5 (26.3)		11 (57.9)	8 (42.1)	
**Country and survey years**							
Cameroon, 2017-2018	13	8 (61.5)	5 (38.5)	.003	4 (30.8)	9 (69.2)	.001
Côte d’Ivoire, 2017-2018	20	8 (40.0)	12 (60.0)		5 (25.0)	15 (75.0)	
Eswatini, 2017-2018	90	73 (81.1)	17 (18.9)		59 (65.6)	31 (34.4)	
Ethiopia, 2017-2018	17	11 (64.7)	6 (35.3)		7 (41.2)	10 (58.8)	
Kenya, 2018	53	39 (73.6)	14 (26.4)		27 (50.9)	26 (49.1)	
Lesotho, 2016-2017	64	50 (78.1)	14 (21.9)		40 (62.5)	24 (37.5)	
Malawi, 2015-2016	86	52 (60.5)	34 (39.5)		42 (48.8)	44 (51.2)	
Namibia, 2017	71	57 (80.3)	14 (19.7)		41 (57.7)	30 (42.3)	
Rwanda, 2018-2019^b^	32	29 (90.6)	3 (9.4)		23 (71.9)	9 (28.1)	
Tanzania, 2016-2017	42	22 (52.4)	20 (47.6)		10 (23.8)	32 (76.2)	
Uganda, 2016-2017	46	22 (47.8)	24 (52.2)		11 (23.9)	35 (76.1)	
Zambia, 2016	41	37 (90.2)	4 (9.8)		21 (51.2)	20 (48.8)	
Zimbabwe, 2015-2016	113	78 (69.0)	35 (31.0)		55 (48.7)	58 (51.3)	

Abbreviations: ART, antiretroviral therapy; CLHIV, children living with HIV; NA, not applicable; PHIA, Population-Based HIV Impact Assessment; VLS, viral load suppression.

^a^ VLS was defined as less than 1000 copies/mL.

^b^ The Rwanda PHIA survey included only children 10 to 14 years of age.

ART uptake and VLS increased with child age: 69 of 142 children (48.6%) aged 0 to 4 years were receiving ART and 39 (27.5%) were virally suppressed compared with 239 of 306 children (78.1%) aged 10 to 14 years receiving ART and 180 (58.8%) with viral suppression. A higher proportion of males than females were receiving ART (246 [73.4%] vs 240 [68.0%]), although VLS was slightly lower in males (160 [47.8%] vs 185 [52.4%]). Maternal age was also associated with each outcome: ART uptake and VLS were lowest among children of mothers aged 15 to 24 years (19 of 35 [54.3%] and 8 [22.9%], respectively) and highest among those with mothers aged 50 to 64 years (14 of 19 [73.7%] and 11 [57.9%], respectively). Maternal engagement was associated with pediatric outcomes. Among children whose mothers were receiving ART, 294 of 357 (82.4%) were receiving ART and 202 (56.6%) achieved VLS compared with 11 of 63 (17.5%) and 10 (15.9%), respectively, among children whose mothers were not receiving ART. Similarly, 258 of 311 children (83.0%) with mothers who had viral suppression were receiving ART, and 185 children (59.5%) had viral suppression. In contrast, 47 of 108 children (43.5%) with mothers who did not have viral suppression were receiving ART and 26 children (24.1%) had VLS.

**Figure 1.**
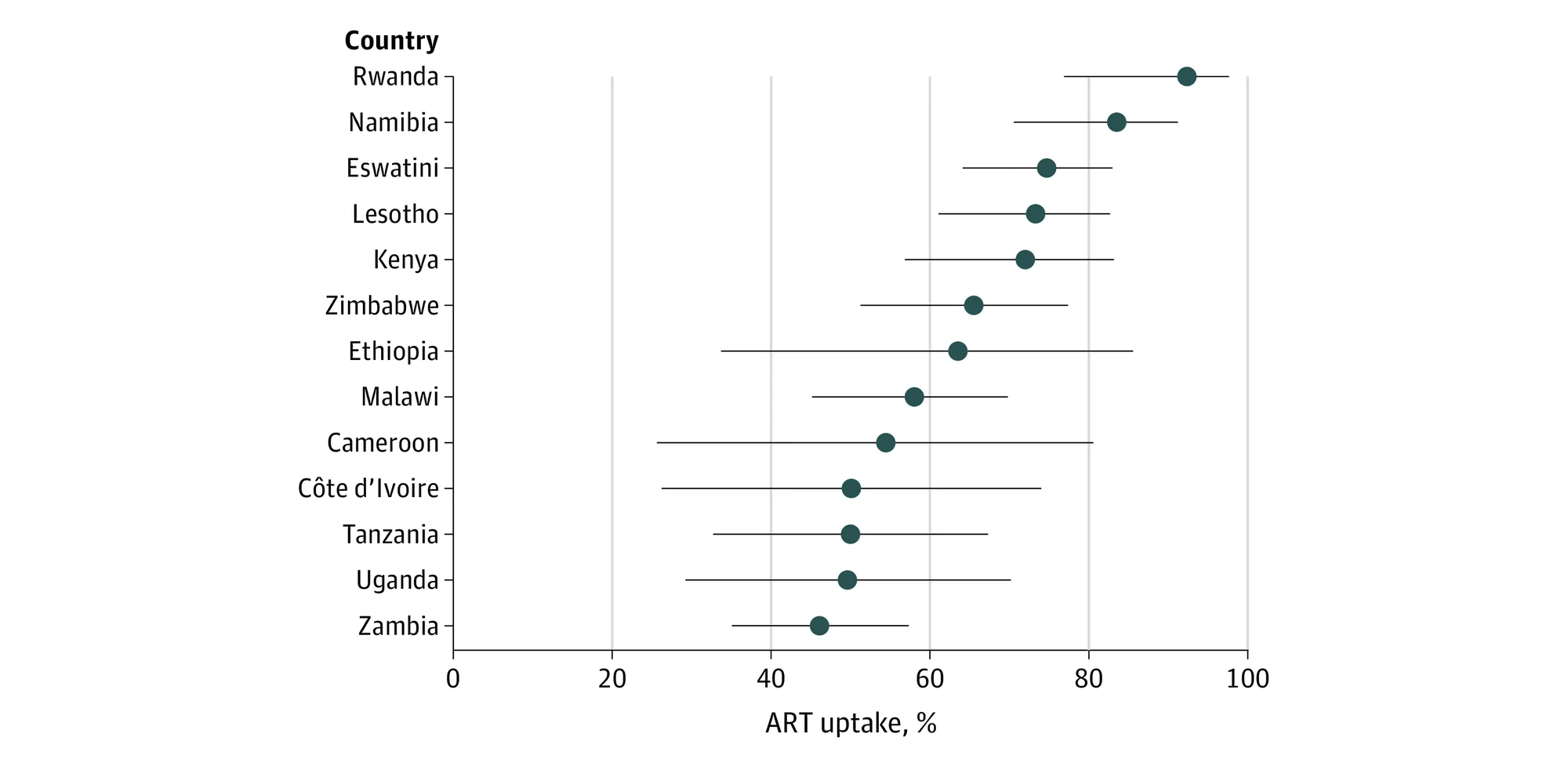
Dot Graph of Prevalence of Antiretroviral Therapy (ART) Uptake Among Children Living With HIV Participating in Population-Based HIV Impact Assessment (PHIA) Surveys From 2015 to 2019 Prevalence estimates were adjusted for the complex survey design. The Rwanda PHIA survey included only children 10 to 14 years of age. Error bars represent 95% CIs.

**Figure 2.**
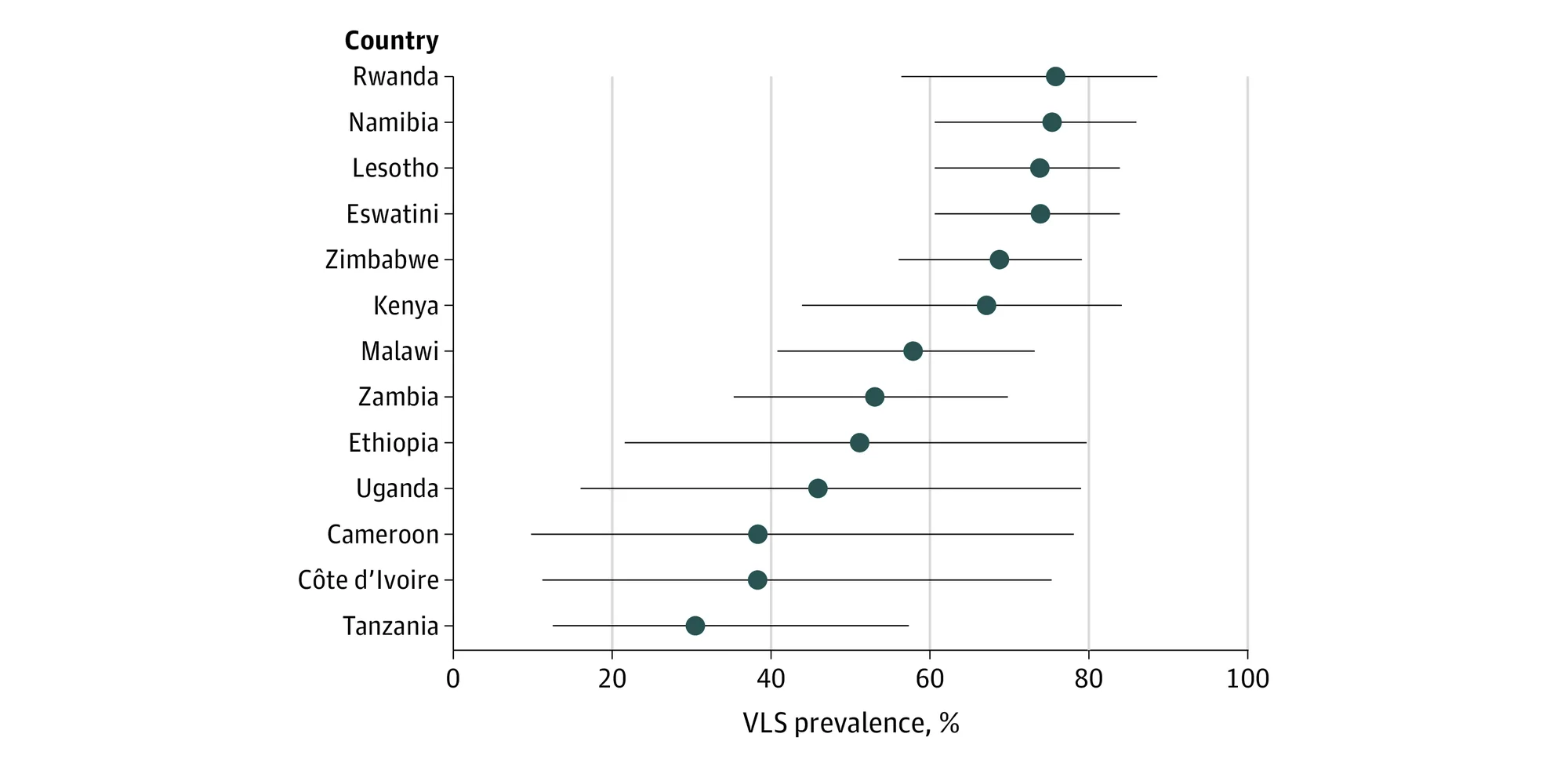
Dot Graph of Viral Load Suppression (VLS) Prevalence Among Children Living With HIV Using Antiretroviral Therapy Participating in Population-Based HIV Impact Assessment (PHIA) Surveys From 2015 to 2019 Prevalence estimates were adjusted for the complex survey design. The Rwanda PHIA survey included only children 10 to 14 years of age. Error bars represent 95% CIs.

Estimated ART uptake is shown in Figure 1. Rwanda had the highest prevalence (92.4%; 95% CI, 77.0%-97.8%), although the sample was restricted to children aged 10 to 14 years. Among surveys including all children younger than 15 years, ART uptake was highest in Namibia (83.5%; 95% CI, 70.7%-91.4%), Eswatini (74.8%; 95% CI, 64.2%-83.1%), and Lesotho (73.4%; 95% CI, 61.4%-82.8%), with Zambia (46.0%; 95% CI, 35.0%-57.5%) and Uganda (49.7%; 95% CI, 29.2%-70.3%) having the lowest prevalence. VLS prevalence (Figure 2) followed similar patterns. Rwanda reported the highest VLS (76.0%; 95% CI, 56.4%-88.6%) among children aged 10 to 14 years. In full pediatric samples, VLS was highest in Namibia (75.4%; 95% CI, 60.7%-85.9%), Lesotho (73.9%; 95% CI, 60.6%-83.9%), and Eswatini (73.9%; 95% CI, 60.6%-83.8%), while Tanzania had the lowest prevalence (30.4%; 95% CI, 12.5%-57.3%).

Restricting the sample exclusively to children with known treatment status (complete-case analysis) shifted the overall pediatric ART uptake prevalence estimate from a primary conservative baseline of 55.6% (95% CI, 49.5%-61.6%) to a complete case of 57.0% (95% CI, 50.8%-62.9%). Complete-case analysis was most consequential for Zambia, where excluding 6 children with unknown status shifted the estimated prevalence of ART uptake from a primary baseline of 46.0% (95% CI, 35.0%-57.5%) to a complete case of 49.2% (95% CI, 37.5%-61.1%) (eTable 8 in Supplement 1). Little difference was observed otherwise.

### Component 2

Table 2 reports multivariable logistic regression results for 686 mother-child dyads. Adjusting for demographic factors and maternal age, maternal ART uptake (model 1a) was significantly associated with child VLS (OR, 3.99; 95% CI, 1.63-9.78); however, this association was attenuated after child ART uptake was included (model 1b: OR, 0.88; 95% CI, 0.30-2.60). Similarly, maternal VLS (model 2a) was significantly associated with child VLS (OR, 3.07; 95% CI, 1.63-5.78) but became nonsignificant after adjusting for child ART uptake (model 2b: OR, 1.66; 95% CI, 0.79-3.48). These primary maternal-child associations remained consistent when restricting the analysis to CLHIV already receiving ART (eTable 2 in Supplement 1) or to mother-child dyads in which the mother was alive and cohabitating (eTable 6 in Supplement 1). Separately, children whose mothers were deceased or had unknown ART status also showed higher VLS than those with mothers who did not have suppression, although these associations were not significant after adjusting for child ART uptake.

**Table 2.** Logistic Regression Models Exploring the Associations Between Maternal Engagement in HIV Care and Viral Load Suppression Among Children Living With HIV

	Outcome: child viral load suppression, OR (95% CI)			
	Maternal ART status		Maternal VLS status	
	Model 1, base adjusted^a^	Model 1 + child ART uptake^b^	Model 2, base adjusted^a^	Model 2 + child ART uptake^b^
Maternal ART status				
No	1 [Reference]	1 [Reference]	NA	NA
Yes	3.99 (1.63-9.78)	0.88 (0.30-2.60)	NA	NA
Alive but with unknown ART status	3.49 (1.35-9.04)	1.15 (0.38-3.48)	NA	NA
Deceased	4.19 (1.60-11.00)	1.27 (0.39-4.19)	NA	NA
Maternal VLS status				
No	NA	NA	1 [Reference]	1 [Reference]
Yes	NA	NA	3.07 (1.63-5.78)	1.66 (0.79-3.48)
Alive but with unknown VLS status	NA	NA	2.53 (1.25-5.12)	2.00 (0.89-4.52)
Deceased	NA	NA	2.96 (1.44-6.11)	2.17 (0.91-5.19)
Maternal age, y				
15-24	0.77 (0.27-2.25)	0.63 (0.22-1.82)	0.71 (0.25-2.06)	0.60 (0.19-1.90)
25-34	1 [Reference]	1 [Reference]	1 [Reference]	1 [Reference]
35-49	1.13 (0.77-1.66)	0.97 (0.62-1.53)	1.15 (0.78-1.70)	0.96 (0.61-1.51)
50-64	1.02 (0.33-3.13)	1.06 (0.15-7.65)	0.92 (0.30-2.83)	1.00 (0.15-6.83)
Child sex				
Male	1 [Reference]	1 [Reference]	1 [Reference]	1 [Reference]
Female	1.30 (0.91-1.86)	1.75 (1.15-2.67)	1.34 (0.93-1.92)	1.77 (1.16-2.69)
Place of residence				
Rural	1 [Reference]	1 [Reference]	1 [Reference]	1 [Reference]
Urban	0.69 (0.45-1.04)	0.62 (0.39-0.98)	0.67 (0.44-1.02)	0.62 (0.39-0.99)
Child age, y				
0-4	1 [Reference]	1 [Reference]	1 [Reference]	1 [Reference]
5-9	2.21 (1.32-3.70)	2.02 (1.09-3.76)	2.34 (1.37-3.98)	1.84 (0.98-3.44)
10-14	2.56 (1.51-4.35)	2.26 (1.19-4.31)	2.05 (1.22-3.45)	2.03 (1.06-3.89)
Child ART status				
No	NA	1 [Reference]	NA	1 [Reference]
Yes	NA	13.89 (7.26-26.59)	NA	12.67 (6.93-23.16)

Abbreviations: ART, antiretroviral therapy; NA, not applicable; OR, odds ratio; VLS, viral load suppression.

^a^ All models accounted for complex survey design matrix settings (stratification, primary sampling units, and population weights). Model 1a shows estimates for maternal ART uptake adjusted for other covariates (n = 686; effective parameters = 13.69; Akaike information criterion [AIC] = 913.4; bayesian information criterion [BIC] = 884.6; C statistic = 0.659; Archer-Lemeshow *F*-adjusted *P* = .72). Model 1b was further adjusted for child ART uptake (n = 686; effective parameters = 17.42; AIC = 784.1; BIC = 827.6; C statistic = 0.789; Archer-Lemeshow *F*-adjusted *P* = .57).

^b^ Model 2a shows estimates for maternal VLS adjusted for other covariates (n = 686; effective parameters = 13.54; AIC = 911.7; BIC = 886.3; C statistic = 0.669; Archer-Lemeshow *F*-adjusted *P* = .98). Model 2b was further adjusted for child ART uptake (n = 686; effective parameters = 17.44; AIC = 780.9; BIC = 824.4; C statistic = 0.794; Archer-Lemeshow *F*-adjusted *P* = .56).

### Component 3

Table 3 shows that both maternal ART uptake (model 3) and maternal VLS (model 4) were associated with child ART uptake. Having a mother receiving ART was associated with higher odds of child ART uptake (OR, 20.42; 95% CI, 8.77-47.54), while maternal VLS was also associated with higher odds of child ART uptake (OR, 5.29; 95% CI, 2.95-9.48).

**Table 3.** Two Logistic Regression Models Exploring the Associations Between Maternal Engagement in HIV Care and ART Uptake Among Children Living With HIV

	Outcome: child ART uptake, OR (95% CI)^a^	
	Maternal ART status	Maternal VLS status
	Model 3^b^	Model 4^c^
Maternal ART status		
No	1 [Reference]	NA
Yes	20.42 (8.77-47.54)	NA
Alive but with unknown ART status	7.98 (3.36-18.96)	NA
Deceased	10.16 (4.11-25.10)	NA
Maternal VLS status		
No	NA	1 [Reference]
Yes	NA	5.29 (2.95-9.48)
Alive but with unknown VLS status	NA	2.08 (1.13-3.84)
Deceased	NA	2.58 (1.30-5.10)
Maternal age, y	NA	
15-24	1.75 (0.55-5.62)	1.44 (0.56-3.72)
25-34	1 [Reference]	1 [Reference]
35-49	1.45 (0.95-2.22)	1.55 (1.01-2.37)
50-64	0.95 (0.19-4.76)	0.80 (0.17-3.90)
Child sex		
Male	1 [Reference]	1 [Reference]
Female	0.61 (0.40-0.93)	0.66 (0.44-0.99)
Place of residence		
Rural	1 [Reference]	1 [Reference]
Urban	1.12 (0.70-1.79)	0.99 (0.64-1.54)
Child age, y		
0-4	1 [Reference]	1 [Reference]
5-9	1.82 (1.01-3.30)	1.76 (0.99-3.13)
10-14	2.18 (1.19-4.00)	2.08 (1.14-3.81)

Abbreviations: ART, antiretroviral therapy; NA, not applicable; VLS, viral load suppression.

^a^ All models accounted for complex survey design matrix settings (stratification, primary sampling units, and population weights).

^b^ Diagnostic and performance metrics are as follows: n = 686; effective parameters = 13.34; Akaike information criterion (AIC) = 718.3; bayesian information criterion (BIC) = 761.4; C statistic = 0.740; Archer-Lemeshow *F*-adjusted *P* = .81.

^c^ Diagnostic and performance metrics are as follows: n = 686; effective parameters = 14.03; AIC = 763.1; BIC = 806.8; C statistic = 0.720; Archer-Lemeshow *F*-adjusted *P* = .72.

Compared with children whose mothers were not receiving ART or had not achieved VLS, children with mothers of unknown ART status or who were deceased had significantly higher odds of receiving ART. Specifically, children of deceased mothers had higher odds of ART uptake in model 3 (OR, 10.16; 95% CI, 4.11-25.10) and in model 4 (OR, 2.58; 95% CI, 1.30-5.10). These findings remained consistent when the sample was restricted to dyads in which the mother was alive and cohabitating (eTable 7 in Supplement 1).

As expected, the association of child ART uptake with child VLS was robust (eTable 3 in Supplement 1). The association between maternal engagement in HIV care and child viral suppression appears to be largely attributed to increased ART uptake. However, there was no evidence that improved VLS among children receiving ART was associated with maternal engagement in HIV care. These results did not change when restricted to mothers who were alive and living in the same household (eTables 6 and 7 in Supplement 1).

## Discussion

Using the PHIA survey data from 13 sub-Saharan countries (2015-2019), we estimated prevalence of ART uptake and VLS among CLHIV and found variation across countries in sub-Saharan Africa. In adjusted logistic regression analysis, we found that maternal engagement in HIV care was associated with VLS among CLHIV. This association was explained by child ART uptake. Mothers receiving ART and mothers with VLS are more likely to have children receiving ART, which substantially increases the likelihood of child VLS. After adjusting for child ART uptake, we did not find evidence that maternal care engagement significantly affected VLS among children. These results highlight the potential benefit of strengthening pediatric ART delivery by supporting family-based care strategies.

Consistent with other studies, we found large variations in ART uptake and VLS among CLHIV between countries.^9^ The variability between countries may be due to, among other things, survey timing vis-à-vis program scale-up, differences in HIV guidelines, and the health system’s ability to deliver accurate and tolerable ART regimens for CLHIV. Although the Rwanda PHIA survey limited its sample to children aged 10 to 14 years, there is also evidence that the country has strengthened its response to the HIV epidemic over the past 2 decades.^36^ Still, observed differences between countries may reflect geographic and demographic variations and unmeasured health system factors. These survey data were collected in 2015 to 2019, before widescale implementation of dolutegravir-based first-line antiretroviral regimens for children,^37^ which have resulted in dramatically increased VLS for children receiving ART^38^ and may have increased ART coverage as a more tolerable regimen.

Other studies have explored caregiver factors associated with child VLS. One study in Kenya found that caregiver membership in a voluntary saving and lending organization may affect child VLS such that children whose caregiver was a member have a greater likelihood of being VLS compared with children whose caregiver was not a member.^21^ Another study based in Ethiopia found that virologic failure in children was significantly associated with family unemployment status such that children receiving ART and living with unemployed family were nearly 5 times more likely to have virological failure.^24^ Our finding that maternal ART uptake and maternal VLS were not independently associated with child VLS after adjusting for child ART status suggests that, while maternal health is important, pediatric ART uptake remains the primary factor in achieving VLS among CLHIV. Nonetheless, identifying the broader social and environmental barriers to pediatric care is essential for developing interventions that move children across the treatment cascade toward VLS.

We found that the overall association between maternal engagement in HIV care and child VLS appeared to operate primarily through child ART uptake. Mothers receiving ART and mothers achieving VLS are much more likely to have children receiving ART, which substantially increases the child’s odds of being virally suppressed. Supporting family-based programs that center caregivers and their children may help initiate CLHIV receiving ART. For example, providing same-day visit for clinically stable caregivers and their children has led to improvement in care for both adult and children.^39^ Other programs including community-based interventions have had promising outcomes. In Lesotho, community intervention groups reported significantly higher HIV testing among children at the 12-month follow-up compared with controls.^40^ National programs should leverage family- and community-based systems to provide the necessary support for mothers and children. By enhancing maternal ART delivery in the context of family- and community-based support systems, national programs may also affect care among CLHIV.

Our analysis also found that children whose mothers had unknown ART uptake or unknown VLS status had higher odds of ART uptake compared with children whose mothers were known to not use ART. One potential explanation is that the unknown category may include mothers who were receiving ART but had incomplete or missing records. Alternatively, children in this group may have benefited from targeted follow-up or interventions due to the lack of maternal information. We also found higher odds of ART uptake among children whose mothers were deceased compared with children whose mothers were not receiving ART. Several studies have found that orphanhood is associated with lower ART uptake and VLS among CLHIV.^41^ However, qualitative evidence suggests that maternal orphans, compared with double orphans, may receive adequate support from grandmothers.^42^ Moreover, a population-based study found high engagement in HIV care among orphans compared with nonorphans.^43^ A growing number of social protection programs have specifically targeted HIV-infected orphans and socially vulnerable children and have been associated with a range of beneficial health outcomes.^44^

This study provides new evidence on how maternal HIV outcomes are associated with child VLS from data spanning several countries across the region most affected by HIV. Routinely explored factors associated with child VLS focus on child characteristics associated with VLS. The multinational population-level estimates provided by the PHIA surveys are strengths of this study, as are VLS data collected and data on maternal HIV outcomes. This multicountry analysis found that the association between maternal HIV care factors and child VLS may operate through child ART uptake. Future studies are needed to explore additional pathways and contextual factors that may affect the relationship between maternal and child HIV outcomes.

### Limitations

This study has several limitations. First, the cross-sectional design limited our ability to establish definitive temporal ordering or causal inference. While our supplementary mediation analysis is highly consistent with an indirect causal pathway whereby maternal engagement in HIV care plays a role in child VLS via the child treatment linkage, cross-sectional data preclude definitive causal inference. Second, potential measurement bias and exposure misclassification may exist. Because ART coverage is estimated among all HIV-positive children, including children with unknown ART status, in settings with substantial missing clinical data, this could bias our coverage estimates toward the null or systematically misclassify child treatment status. Third, survey design relies on household dyadic linking, which serves as a proxy and does not definitively confirm whether the women identified in the mother-child dyads were mainly responsible for HIV management and care for the child in all instances. However, our sensitivity analysis, which included sample restriction to dyads who lived in the same household, making the maternal-child link more plausible, found similar results. Fourth, although we adjusted for several key sociodemographic factors, there may be unmeasured confounding from unobserved factors, such as individual ART adherence, therapy duration, medication regimen, and the broader caregiving environment, that could change the observed associations. Finally, the regression analysis was restricted to children with known ART and VLS status, limiting generalizability and potentially leading to bias estimates if missingness is not random.

## Conclusions

In this cross-sectional study of PHIA survey data, we found persistent disparities in VLS among CLHIV across sub-Saharan Africa. Despite decades of progress in HIV treatment, a substantial gap in care equity remains between children and adults living with HIV, with children often experiencing disproportionately high mortality rates. The results underscore the importance of health system investment in linkage and retention strategies for mothers and CLHIV to maximize the impact of ART programs.

Child VLS is complicated by the heavy dependence of ART uptake and adherence on caregiver involvement. By exploring caregiver contexts—specifically caregiver engagement in HIV care—we may gain a more nuanced understanding of VLS among children, which could ultimately help achieve the third 95 (ie, VLS) of the UNAIDS 95-95-95 HIV treatment targets for children. Our finding that maternal ART use and VLS status are associated with child VLS, indirectly through its role in child ART uptake, provides important insight into the critical role of maternal engagement in care in child outcomes. These findings should not be interpreted as diminishing the importance of maternal viral suppression to maternal health or transmission prevention but rather as highlighting the central role of timely ART initiation and retention for children.

## Notes

Supplement 1. **eMethods 1.** PHIA Survey Details **eTable 1.** PHIA Survey Sampling Strategy for Children in 13 PHIA Surveys (2015-2019) **eMethods 2.** Secondary Analysis Sample Selection **eFigure 1.** Description of Analytic Sample for Each Step in the Secondary Analysis **eFigure 2.** Distribution of Viral Load Suppression Among Children Living With HIV Across Survey Sample (Full Survey Data), Descriptive Sample, and Children With Missing Parent Information **eMethods 3.** Analytic Variables **eMethods 4.** Model Specifications **eMethods 5.** Sensitivity Analysis: CLHIV on ART **eTable 2.** Logistic Regression From Models Exploring Associations Between Maternal Engagement in HIV Care Factors and Viral Load Suppression (VLS) Among All Children (<15 Years of Age) Living With HIV on ART **eFigure 3.** Conceptual Framework Linking Maternal Engagement in HIV Care Factors (ART Uptake and VLS) to Key Steps in the Pediatric HIV Care Cascade: Child ART Uptake and Child VLS **eTable 3.** Logistic Regression Model Exploring the Association Between Child ART Uptake and Child VLS Among All Children (<15 Years of Age) Living With HIV **eMethods 6.** Alternative Analysis: Mediation Analysis **eTable 4.** Decomposition of Maternal Total Effects on Child Viral Load Suppression via Child ART Uptake (n = 419) **eTable 5.** Assessment of Collinearity Among Predictors in Multivariable Logistic Regression Models via Generalized Variance Inflation Factors (GVIF) **eMethods 7.** Sensitivity Analysis: Mother-Child Dyads in the Same Household **eTable 6.** Logistic Regression From 2 Separate Models Exploring Associations Between Maternal Engagement in HIV Care Factors and VLS Among All Children (<15 Years of Age) Living With HIV **eTable 7.** Logistic Regression From Two Separate Models Exploring Maternal Factors Associated With ART Uptake Among All Children (<15 Years of Age) Living With HIV **eTable 8.** Comparison of Primary Prevalence Estimates and Prevalence From Complete-Case Sensitivity Analysis
eReferences

Supplement 2.
Data Sharing Statement
