# Supplementary material for: Maternal Engagement in HIV Care and Antiretroviral Therapy Uptake Among Children Living With HIV
Source: JAMA Netw Open. 2026 Aug 26;9(8):e2630983. doi: 10.1001/jamanetworkopen.2026.30983 (PMC13519847; doi:10.1001/jamanetworkopen.2026.30983)
Supplement: Supplementary file 1 [file jamanetwopen-e2630983-s001.pdf]

Supplemental Online Content

He J, Trejo BG, Oumer KS, et al. Maternal engagement in HIV care and antiretroviral therapy uptake among children living with HIV. *JAMA Netw Open*. 2026;9(8):e2630983.
doi:10.1001/jamanetworkopen.2026.30983

eMethods 1. PHIA Survey Details

eTable 1. PHIA Survey Sampling Strategy for Children in 13 PHIA Surveys (2015-2019)

eMethods 2. Secondary Analysis Sample Selection

eFigure 1. Description of Analytic Sample for Each Step in the Secondary Analysis

eFigure 2. Distribution of Viral Load Suppression Among Children Living With HIV Across Survey Sample (Full Survey Data), Descriptive Sample, and Children With Missing Parent Information

eMethods 3. Analytic Variables

eMethods 4. Model Specifications

eMethods 5. Sensitivity Analysis: CLHIV on ART

eTable 2. Logistic Regression From Models Exploring Associations Between Maternal Engagement in HIV Care Factors and Viral Load Suppression (VLS) Among All Children (<15 Years of Age) Living With HIV on ART

eFigure 3. Conceptual Framework Linking Maternal Engagement in HIV Care Factors (ART Uptake and VLS) to Key Steps in the Pediatric HIV Care Cascade: Child ART Uptake and Child VLS

eTable 3. Logistic Regression Model Exploring the Association Between Child ART Uptake and Child VLS Among All Children (<15 Years of Age) Living With HIV

eMethods 6. Alternative Analysis: Mediation Analysis

eTable 4. Decomposition of Maternal Total Effects on Child Viral Load Suppression via Child ART Uptake (n = 419)

© 2026 He J et al. *JAMA Netw Open*.

eTable 5. Assessment of Collinearity Among Predictors in Multivariable Logistic Regression Models via Generalized Variance Inflation Factors (GVIF)

eMethods 7. Sensitivity Analysis: Mother-Child Dyads in the Same Household

eTable 6. Logistic Regression From Two Separate Models Exploring Associations Between Maternal Engagement in HIV Care Factors and VLS Among All Children (<15 Years of Age) Living With HIV

eTable 7. Logistic Regression From 2 Separate Models Exploring Maternal Factors Associated With ART Uptake Among All Children (<15 Years of Age) Living With HIV

eTable 8. Comparison of Primary Prevalence Estimates and Prevalence From Complete-Case Sensitivity Analysis

eReferences

This supplemental material has been provided by the authors to give readers additional information about their work.

© 2026 He J et al. *JAMA Netw Open*.

eMethods 1. PHIA survey details

1.1 PHIA survey

PHIA surveys consisted of a household questionnaire completed by the head of household or designee, individual questionnaire completed by consenting individual participants, point-of-care HIV serologic testing, and laboratory HIV-related biomarker testing. Parents and guardians answered questions about their children's health and participation in HIV testing and care services. Children were eligible to participate if a parent or guardian provided consent or permission. Older minors (defined separately by each country) were asked to provide written assent after parent/guardian informed consent. Samples for all participants who tested positive for HIV with rapid point-of-care tests during the survey were laboratory confirmed using the Geenius HIV 1/2 supplemental assay.[1] During the survey, viral load testing and ART detection were conducted among HIV-positive children in a laboratory. Detection of ARTs indicates participant use of a given drug at the time of blood collection and was quantified by qualitative screening which was conducted on blood specimens from all HIV-positive children, using high-resolution liquid chromatography coupled with tandem mass spectrometry.

PHIA survey protocols including recruitment material, consent forms, and questionnaires were reviewed and approved by relevant national ethics and regulatory bodies and the institutional review boards (IRBs) of Columbia University Medical Center, Westat, and the U.S. Centers for Disease Control and Prevention.

1.2 PHIA survey child sample selection

The following PHIA surveys were included in this analysis: Cameroon (2017-2018),[2] Côte d'Ivoire (2017-2018),[3] Eswatini (2016-2017),[4] Ethiopia (2017-2018),[5] Kenya (2018),[6] Lesotho (2016-2017),[7]

Malawi (2015-2016),[8] Namibia (2017),[9] Rwanda (2018-2019),[10] Tanzania (2016-2017),[11] Uganda (2016-2017),[12] Zambia (2016),[13] and Zimbabwe (2015-2016).[14]

All surveys were cross-sectional, household-based, two-stage cluster sample surveys designed to measure HIV program impact and capture the state of the HIV epidemic among adults aged 15–64 years and children 0–14 years of age (with variations by survey). eTable 1 indicates differences in sampling strategy for children by survey country.

© 2026 He J et al. *JAMA Netw Open*.

eTable 1: PHIA survey sampling strategy for children in 13 PHIA surveys (2015-2019)

Description of sampling strategy	Survey country	Notes
Eligible children from one third of sampled households	Cameroon, Kenya, Tanzania	
Eligible children from one half sampled households	Eswatini, Ethiopia, Lesotho, Malawi, Namibia, Zambia, Zimbabwe	
Eligible children from all sampled household	Uganda	All eligible children 0-14 years of age in randomly designated households were included in the study for data collection, with children 0-4 years of age selected at higher rates than children 5-14 years of age. (Differential selection probability accounted for in survey weights.)
Other	Côte d'Ivoire	Children 0-14 years of age were to be subsampled for blood testing at rates that depended on the HIV status of their mother. If the child's mother had tested positive for HIV, was deceased, or whose HIV status was unknown, the child was designated as "targeted" and sampled with certainty for blood testing.
	Rwanda	Children 0-9 years old were excluded from the study due to the very low rates of HIV infection among this age group. Within the sampled households, all eligible persons 10 to 64 years of age were included in the study sample for PHIA data collection.

© 2026 He J et al. *JAMA Netw Open*.

eMethods 2. Secondary analysis sample selection

2.1 Study analytic sample selection

We analyzed data on children living with HIV with known ART and VLS status. A total of 728 children with known ART and VLS status were included in the combined survey samples. The analytic sample for the descriptive statistics (component 1) included 688 children living with HIV with maternal data. For the regression analysis (component 2 & 3) we restricted the sample to 686 mother-child dyads where the mother survival status was known . Finally, for the sensitivity analysis, we further excluded mother-child dyads in which the mother was deceased or was not living in the same household, yielding an analytic sample for the sensitivity analysis of 431 mother-child dyads. eFigure 1 outlines the different sample sizes across the different analysis.

© 2026 He J et al. *JAMA Netw Open*.

eFigure 1: Description of analytic sample for each step in the secondary analysis

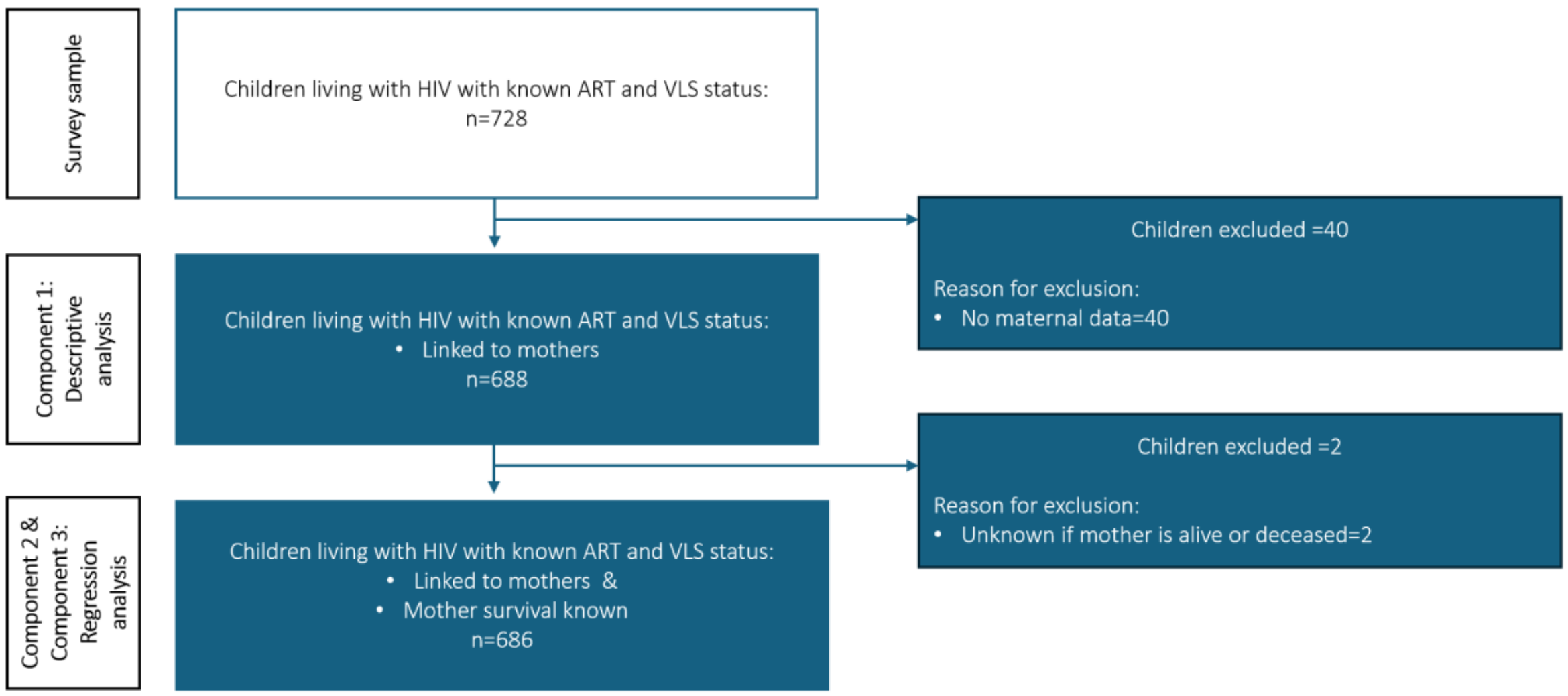

© 2026 He J et al. *JAMA Netw Open*.

2.2 Comparison between survey sample (n=728) and descriptive analysis (n=688)

For the descriptive analysis (component 1) of this secondary study, we limited the sample to children with maternal data, irrespective of maternal survival and survey participation. As a result, data for 40 children were excluded. The differences in ART uptake and VLS between the full survey sample and this restricted sample are shown in eFigure 2 below.

eFigure 2. Distribution of viral load suppression among children living with HIV across survey sample (full survey data), descriptive sample, and children with missing parent information.

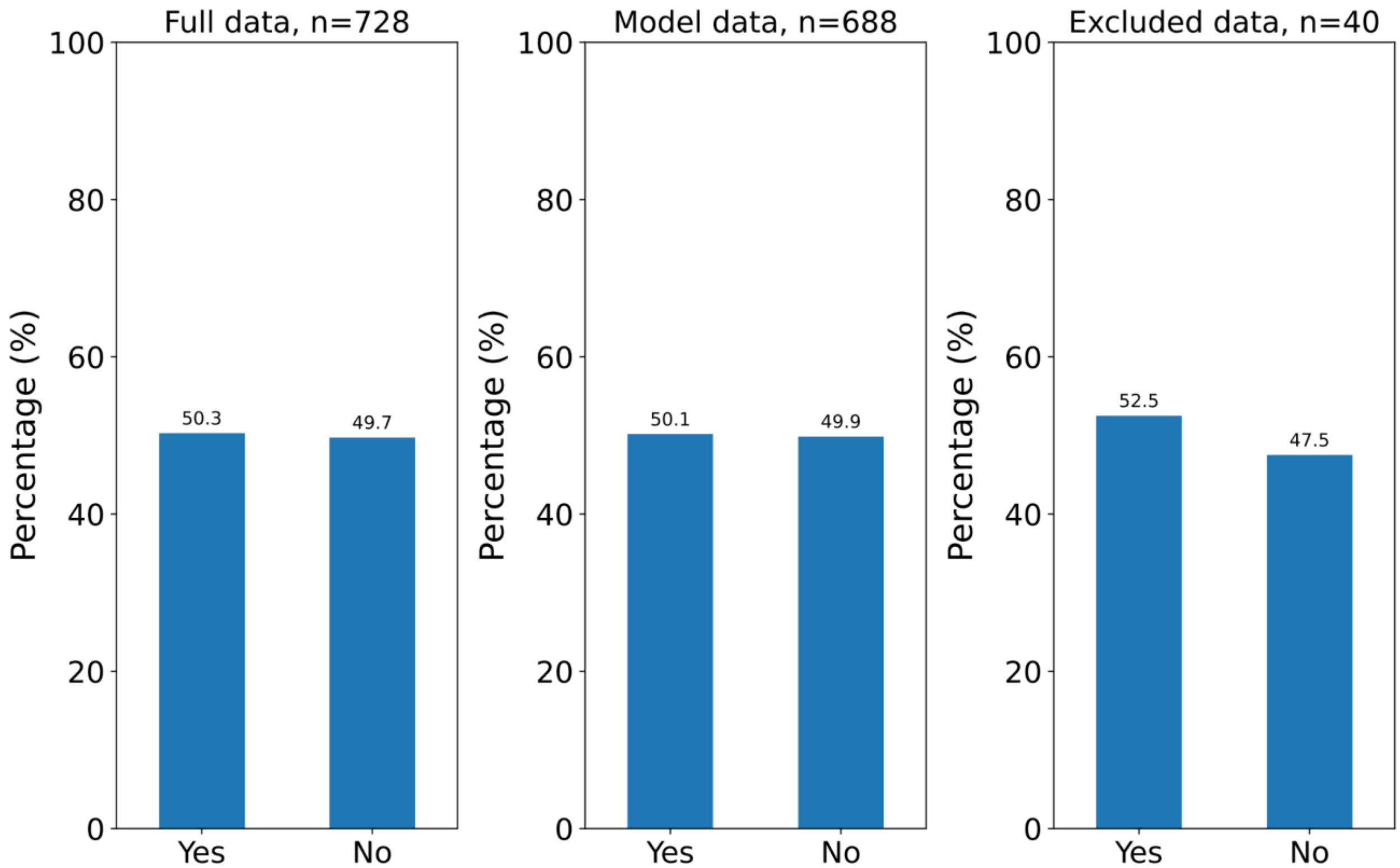

© 2026 He J et al. *JAMA Netw Open*.

eMethods 3. Analytic variables

3.1 Antiretroviral therapy (ART) status

Child ART status was categorized as yes (on ART) or no (not on ART) using PHIA survey variables *pedart* which combined biomarker detection status in an individual's blood sample and caregiver reported. Children with *pedart*=1 (ARVs detectable, parent reported on ART, or both ARVs detectable and parent-reported on ART) were categorized as *yes*. Children with *pedart*=2 were categorized as *no*. Children with missing values for *pedart* were included in the denominator for the prevalence estimations but were otherwise excluded from the regression analysis.

Maternal ART status was categorized as yes (on ART), no (not on ART), unknown (mother alive—ART status not known), or mother deceased using PHIA survey variable *arvstatus*. Mothers with *arvstatus*=1 (ARVs detected) were categorized as *yes*. Mothers with *arvstatus*=2 were categorized as *no*. Mothers with missing values for *arvstatus* were categorized as "alive, ART status unknown", "deceased", or "survival status unknown" using child survey variable *momalive*.

3.2 Viral load suppression (VLS) status

Maternal and child VLS status was categorized as "suppressed" and "unsuppressed" using PHIA survey variable *vls* which follows WHO guidelines that define viral load suppressed as less than 1000 copies/ml and unsuppressed viral load as at least 1000 copies/ml. Children with missing values were excluded from the regression analysis. Mothers with missing values for vls were categorized as "alive, VLS status unknown", "deceased", or "survival status unknown" using child survey variable *momalive*.

© 2026 He J et al. *JAMA Netw Open*.

eMethods 4. Model specifications

Model 1a and 2a were logistic regression models for the effect of maternal engagement in HIV care and child VLS adjusting for pre-specified covariates:

$$logit\big(P(Y = 1 \mid X, C)\big) = \beta_0 + \beta_1 X + \beta_2 C$$

Model 1b and 2b extend Model 1a and Model 2a by further adjusting for child ART uptake:

$$logit\big(P(Y = 1 \mid X, C, M)\big) = \beta_0 + \beta_1 X + \beta_2 C + \beta_3 M$$

In these models, **X** denotes the maternal engagement in HIV care variables (maternal ART uptake in model 1b and maternal VLS in model 2b), **Y** is the outcome (child VLS), **C** represents the vector of covariates, and **M** represents child on ART status.

Model 3 & 4, were logistic regression models for the effect of maternal engagement in HIV care and child being on ART adjusting for pre-specified covariates:

$$logit\big(P(M = 1 \mid X, C)\big) = \beta_0 + \beta_1 X + \beta_2 C$$

In this model, **M** is the outcome (child on ART), while β_1 measures the effect of maternal exposure on the probability of a child being on ART, adjusting for covariates.

© 2026 He J et al. *JAMA Netw Open*.

eMethods 5. Sensitivity analysis: CLHIV on ART

The following results modify model 1b (maternal ART uptake) and 2b (maternal VLS) by exploring the association between these maternal factors and child VLS only among CLHIV on ART (n=484).

eTable 2: Logistic regression from models exploring associations between maternal engagement in HIV care factors and viral load suppression (VLS) among all children (<15 years of age) living with HIV on ART.

	Model 1b	Model 2b
**Maternal ART status**		
No	Ref	
Yes	0.72 (0.11-4.99)	
Mother alive—ART status unknown	1.05 (0.15-7.56)	
Mother deceased	1.01 (0.14-7.37)	
**Maternal VLS status**		
No		Ref
Yes		1.68 (0.71-3.98)
Mother alive—VLS status unknown		2.23 (0.86-5.76)
Mother deceased		2.17 (0.81-5.79)
**Child sex**		
Male	Ref	Ref
Female	1.76 (1.11-2.80)	1.77 (1.11-2.81)
**Place of Residence**		
Rural	Ref	Ref
Urban	0.62 (0.38-1.03)	0.63 (0.38-1.05)
**Child Age**		
0-4 years	Ref	Ref
5-9 years	2.46 (1.24-4.87)	2.23 (1.13-4.44)
10-14 years	2.63 (1.33-5.22)	2.36 (1.19-4.71)
**Maternal Age**		
15-24 years	0.76 (0.23-2.48)	0.74 (0.20-2.80)
25-34 years	Ref	Ref
35-49 years	0.97 (0.59-1.59)	0.96 (0.58-1.58)
50-64 years	0.55 (0.12-2.50)	0.54 (0.12-2.40)

Abbreviations: AIC, Akaike Information Criterion; BIC, Bayesian Information Criterion.
a All models account for complex survey design matrix settings (stratification, primary sampling units, and population weights).
b Diagnostic and performance metrics:

- **Model 1b: N = 484; Effective Parameters = 14.76; AIC = 628.1; BIC = 666.6; C-Statistic = 0.659; Archer–Lemeshow F-adjusted P = 0.933**
- **Model 2b: N = 484; Effective Parameters = 14.53; AIC = 625.6; BIC = 664.6; C-Statistic = 0.672; Archer–Lemeshow F-adjusted P = 0.367**

© 2026 He J et al. *JAMA Netw Open*.

Conceptual framework

eFigure 3: Conceptual framework linking maternal engagement in HIV care factors (ART uptake and VLS) to key steps in the pediatric HIV care cascade: child ART uptake and child VLS.

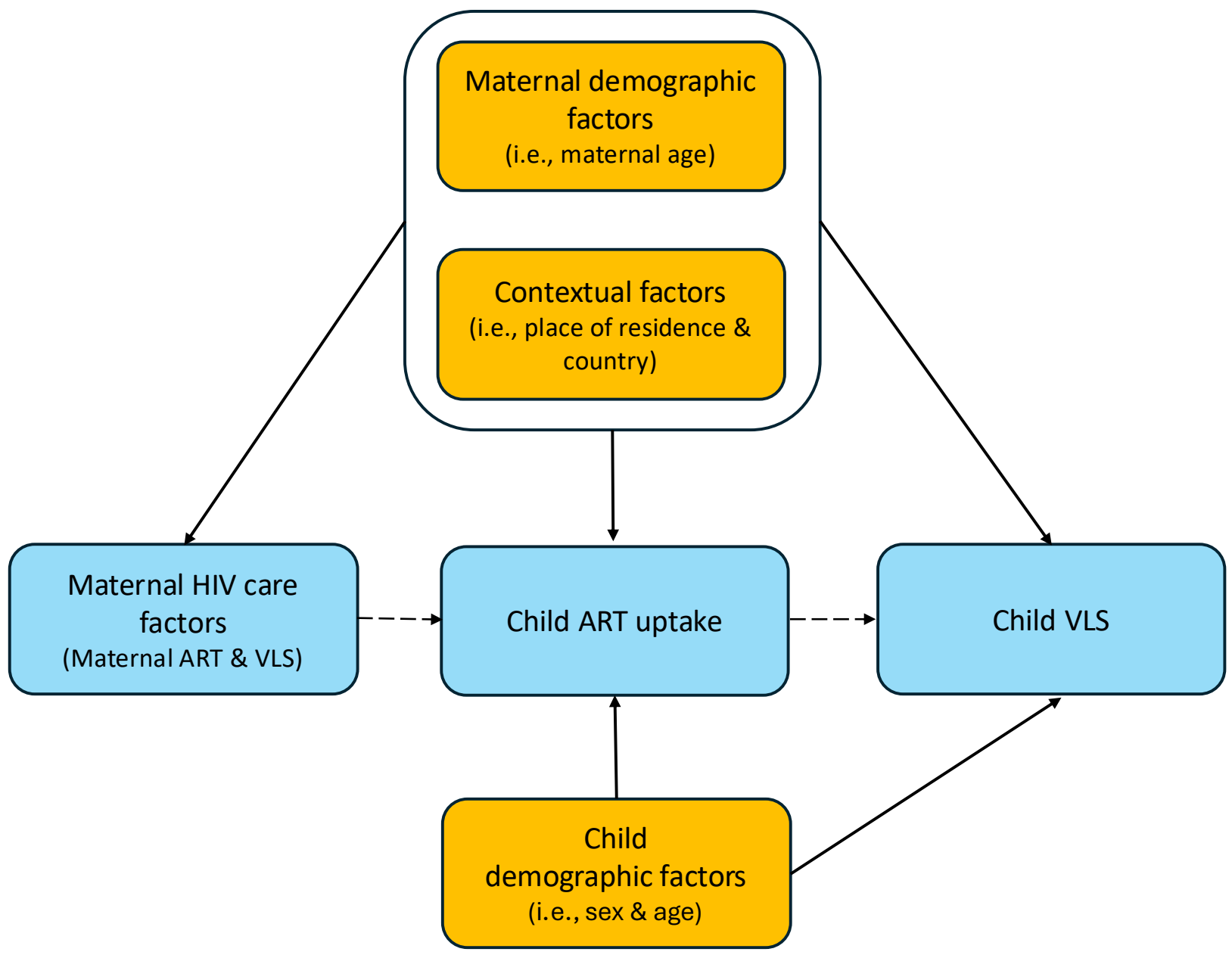

© 2026 He J et al. *JAMA Netw Open*.

Association between child ART uptake and child VLS

eTable 3: Logistic regression model exploring the association between child ART uptake and child VLS among all children (<15 years of age) living with HIV.

	OR (95% confidence interval)
**Child's ART status**	
No	Ref
Yes	12.22 (5.62-26.55)
**Child's sex**	
Male	Ref
Female	2.36 (1.33-4.17)
**Place of residence**	
Rural	Ref
Urban	1.14 (0.62-2.08)
**Child's age**	
0-4 years of age	Ref
5-9 years of age	1.79 (0.76-4.20)
10-14 years of age	2.43 (0.98-5.98)
**Mother's age**	
15-24 years of age	0.75 (0.18-3.18)
25-34 years of age	Ref
35-49 years of age	1.24 (0.68-2.27)
50-64 years of age	2.67 (0.22-33.07)

© 2026 He J et al. *JAMA Netw Open*.

eMethods 6. Alternative analysis: Mediation analysis

To evaluate the underlying mechanisms linking maternal engagement to pediatric clinical outcomes, we conducted a formal causal mediation analysis. This analysis decomposes the Total Effect of maternal engagement into two distinct pathways:

(1) Indirect effect (Mediation Pathway): The effect of the maternal exposure on child VLS that acts through the intermediate step of child ART uptake.

(2) Direct effect: The remaining effect of the maternal exposure on child VLS that operates independently of child ART uptake.

The mediation analysis was restricted to a subpopulation of 419 mother-child dyads with complete, known data for both maternal ART and VLS status. Because the underlying models are logistic regressions mapping binary outcomes, we utilized the product-of-coefficients (Sobel) method calculated on the logit scale. Variance and 95% confidence intervals (CIs) for the indirect and direct effects were derived analytically using the delta method.[15,16]

Given the cross-sectional design of the pooled PHIA data, these pathways are framed as structurally consistent with—rather than definitive longitudinal proof of—an indirect causal pathway.

For both maternal ART uptake and VLS, the total association with pediatric viral load suppression was significantly driven by the indirect pathway through child ART uptake.

© 2026 He J et al. *JAMA Netw Open*.

eTable 4: Decomposition of maternal total effects on child viral load suppression via child ART uptake (n = 419)

Exposure	effect	β on logit scale	95% Confidence Intervals	p value	Proportion mediated (%)
Maternal ART uptake	Total	8.3	4.709 to 11.89	<0.001	101%
	Direct	-0.081	-1.193 to 1.031	0.89	
	Indirect (via child ART)	8.381	4.967 to 11.795	<0.001	
Maternal VLS	Total	4.878	2.815 to 6.942	<0.001	88%
	Direct	0.573	-0.177 to 1.324	0.13	
	Indirect (via child ART)	4.305	2.383 to 6.227	<0.001	

© 2026 He J et al. *JAMA Netw Open*.

Generalized variance inflation factor (GVIF) diagnostics

eTable 5: Assessment of collinearity among predictors in multivariable logistic regression models via generalized variance inflation factors (GVIF).

model	Predictor	GVIF	Degrees of Freedom (*df*)	adjusted GVIF
Model 1a	Child's sex	1.07	1	1.04
Model 1a	Place of residence	1.05	1	1.03
Model 1a	Child's age group	1.11	2	1.03
Model 1a	Mother's age group	1.35	3	1.05
Model 1a	Mother's ART status	1.33	3	1.05
Model 1b	Child's sex	1.13	1	1.06
Model 1b	Place of residence	1.04	1	1.02
Model 1b	Child's age group	1.42	2	1.09
Model 1b	Mother's age group	1.51	3	1.07
Model 1b	Mother's ART status	1.61	3	1.08
Model 1b	Child's ART status	1.35	1	1.16
Model 2a	Child's sex	1.07	1	1.04
Model 2a	Place of residence	1.05	1	1.02
Model 2a	Child's age group	1.12	2	1.03
Model 2a	Mother's age group	1.29	3	1.04
Model 2a	Mother's VLS status	1.27	3	1.04
Model 2b	Child's sex	1.13	1	1.06
Model 2b	Place of residence	1.04	1	1.02
Model 2b	Child's age group	1.39	2	1.09
Model 2b	Mother's age group	1.49	3	1.07
Model 2b	Mother's VLS status	1.43	3	1.06
Model 2b	Child's ART status	1.16	1	1.07
Model 3	Child's sex	1.03	1	1.02
Model 3	Place of residence	1.09	1	1.04
Model 3	Child's age group	1.8	2	1.16
Model 3	Mother's age group	2.25	3	1.14
Model 3	Mother's ART status	1.42	3	1.06
Model 4	Child's sex	1.03	1	1.01
Model 4	Place of residence	1.1	1	1.05
Model 4	Child's age group	1.88	2	1.17
Model 4	Mother's age group	2.16	3	1.14
Model 4	Mother's VLS status	1.39	3	1.06

© 2026 He J et al. *JAMA Netw Open*.

eMethods 7. Sensitivity analysis: mother-child dyads in the same household

Component 2: Maternal engagement in HIV care (ART uptake & VLS) and child VLS

The PHIA survey data does not definitively confirm whether the women identified in the mother-child dyads were responsible for HIV management and care for the child in all instances. To more plausibly proxy this relationship, we restricted the sample to dyads where the mother was alive and lived in the same household (n=431). Sensitivity analysis results for component 2 is presented below:

© 2026 He J et al. *JAMA Netw Open*.

eTable 6: Logistic regression from two separate models exploring associations between maternal engagement in HIV care factors and VLS among all children (<15 years of age) living with HIV.

	**Child VLS**	
	**Model 1b**	**Model 2b**
	**OR (95% Confidence interval)**	**OR (95% Confidence interval)**
**Maternal ART status**		
No	Ref	
Yes	1.38 (0.40-4.74)	
Unknown	1.27 (0.32-5.14)	
**Maternal VLS status**		
No		Ref
Yes		1.82 (0.84-3.96)
Unknown		1.62 (0.60-4.40)
**Child sex**		
Male	Ref	Ref
Female	1.86 (1.12-3.07)	1.90 (1.15-3.15)
**Place of Residence**		
Rural	Ref	Ref
Urban	0.80 (0.47-1.36)	0.79 (0.46-1.36)
**Child Age**		
0-4 years	Ref	Ref
5-9 years	2.11 (1.00-4.42)	1.90 (0.88-4.08)
10-14 years	2.42 (1.09-5.39)	2.13 (0.93-4.88)
**Maternal Age**		
15-24 years	0.81 (0.26-2.50)	0.74 (0.22-2.42)
25-34 years	Ref	Ref
35-49 years	1.06 (0.60-1.85)	1.04 (0.59-1.83)
50-64 years	1.29 (0.22-7.70)	1.20 (0.21-6.91)
**Child ART status**		
No	Ref	Ref
Yes	8.74 (4.13-18.50)	8.50 (4.28-16.91)

<u>Component 3: Linking maternal engagement in HIV care (ART uptake and VLS) and child HIV care cascade</u>

The PHIA survey data does not definitively confirm whether the women identified in the mother-child dyads were responsible for HIV management and care for the child in all instances. To more plausibly proxy this relationship, we restricted the sample to dyads where the mother was alive and lived in the same household, and where maternal ART uptake and VLS status was known (n=355). Sensitivity analysis results for component 3 of the main analysis is presented below:

eTable 7: Logistic regression from 2 separate models exploring maternal factors associated with ART uptake among all children (<15 years of age) living with HIV.

	**Child ART uptake**	
	**Model 3**	**Model 4**
	**OR (95% confidence interval)**	**OR (95% confidence interval)**
**Maternal ART status**		
No	Ref	
Yes	23.98 (9.14-62.96)	
**Maternal VLS status**		
No		Ref
Yes		4.89 (2.66-8.99)
**Child's sex**		
Male	Ref	Ref
Female	0.40 (0.21-0.76)	0.48 (0.27-0.85)
**Place of residence**		
Rural	Ref	Ref
Urban	1.12 (0.58-2.17)	0.90 (0.50-1.62)
**Child's age**		
0-4 years of age	Ref	Ref
5-9 years of age	1.50 (0.66-3.39)	1.51 (0.70-3.29)
10-14 years of age	2.53 (1.02-6.28)	2.58 (1.06-6.27)
**Mother's age**		
15-24 years of age	2.35 (0.35-15.97)	1.52 (0.43-5.32)
25-34 years of age	Ref	Ref
35-49 years of age	1.30 (0.68-2.50)	1.43 (0.75-2.72)
50-64 years of age	0.42 (0.08-2.21)	0.31 (0.07-1.50)

© 2026 He J et al. *JAMA Netw Open*.

Sensitivity analysis: Complete case prevalence estimates

eTable 8: Comparison of primary ART prevalence estimates and ART prevalence from complete-case sensitivity analysis

Country	Number HIV positive	Number with unknown ART	Primary prevalence	Prevalence excluding those with unknown ART
**Cameroon**	14	0	54.5 (25.6-80.7)	54.5 (25.6-80.7)
**Côte d'Ivoire**	23	0	50.2 (26.2-74.1)	50.2 (26.2-74.1)
**Eswatini**	96	0	74.8 (64.2-83.1)	74.8 (64.2-83.1)
**Ethiopia**	17	0	63.6 (33.7-85.7)	63.6 (33.7-85.7)
**Kenya**	57	0	72.0 (56.9-83.3)	72.0 (56.9-83.3)
**Lesotho**	84	2	73.4 (61.4-82.8)	74.9 (63.0-84.0)
**Malawi**	99	0	58.1 (45.2-69.9)	58.1 (45.2-69.9)
**Namibia**	74	0	83.5 (70.7-91.4)	83.5 (70.7-91.4)
**Rwanda**	33	0	92.4 (77.0-97.8)	92.4 (77.0-97.8)
**Tanzania**	45	0	50.1 (32.7-67.4)	50.1 (32.7-67.4)
**Uganda**	52	3	49.7 (29.2-70.3)	54.2 (32.7-74.3)
**Zambia**	95	6	46.0 (35.0-57.5)	49.2 (37.5-61.1)
**Zimbabwe**	120	0	65.6 (51.3-77.6)	65.6 (51.3-77.6)
**OVERALL (pooled)**	809	11	55.6 (49.5-61.6)	57.0 (50.8-62.9)

Note: ART uptake prevalence was calculated among all children living with HIV, irrespective of whether they had VLS data.

© 2026 He J et al. *JAMA Netw Open*.

eReferences

1. Patel HK, Duong YT, Birhanu S, et al. A Comprehensive Approach to Assuring Quality of Laboratory Testing in HIV Surveys: Lessons Learned From the Population-Based HIV Impact Assessment Project. *J Acquir Immune Defic Syndr*. 2021;87(Suppl 1):S17-S27. doi:10.1097/QAI.0000000000002702

2. Ministry of Public Health Cameroon, ICAP at Columbia University. Cameroon Population-based HIV Impact Assessment (CAMPHIA), 2017-2018. Published online 2020. https://phia-data.icap.columbia.edu

3. Ministère de la Santé et de l'Hygiène Publique (MSHP), Institut National de la Statistique (INS), ICAP at Columbia University, and US Centers for Disease Control and Prevention (CDC). Côte d'Ivoire Population-based HIV Impact Assessment (CIPHIA), 2017-2018. https://phia-data.icap.columbia.edu/datasets?country_id=13

4. Ministry of Health (MOH) [Eswatini], Central Statistical Office (CSO), ICAP at Columbia University, and US Centers for Disease Control and Prevention (CDC). Swaziland HIV Incidence Measurement Survey 2 (SHIMS2), 2016–2017. https://phia-data.icap.columbia.edu/datasets?country_id=2

5. Ethiopian Public Health Institute (EPHI), Federal HIV/AIDS Prevention and Control Office (HAPCO), ICAP at Columbia University, and US Centers for Disease Control and Prevention (CDC). Ethiopia Population-based HIV Impact Assessment (EPHIA), 2017–2018. https://phia-data.icap.columbia.edu/datasets?country_id=12

6. Ministry of Health (MOH) [Kenya], National AIDS and STI Control Programme (NASCOP), Kenya National Bureau of Statistics (KNBS), ICAP at Columbia University, and US Centers for Disease Control and Prevention (CDC). Kenya Population-based HIV Impact Assessment (KENPHIA), 2018. https://phia-data.icap.columbia.edu/datasets?country_id=14

7. Ministry of Health (MOH) [Lesotho], Lesotho Bureau of Statistics (BOS), ICAP at Columbia University, and US Centers for Disease Control and Prevention (CDC). Lesotho Population-based HIV Impact Assessment (LePHIA), 2016–2017. https://phia-data.icap.columbia.edu/datasets?country_id=8

8. Ministry of Health (MOH) [Malawi], ICAP at Columbia University, and US Centers for Disease Control and Prevention (CDC). Malawi Population-based HIV Impact Assessment (MPHIA), 2015–2016. https://phia-data.icap.columbia.edu/datasets?country_id=3

© 2026 He J et al. *JAMA Netw Open*.

9. Ministry of Health and Social Services (MoHSS) [Namibia], Namibia Statistics Agency (NSA), ICAP at Columbia University, and US Centers for Disease Control and Prevention (CDC). Namibia Population-based HIV Impact Assessment (NAMPHIA), 2017. https://phia-data.icap.columbia.edu/datasets?country_id=9

10. Rwanda Ministry of Health (MOH), Rwanda Biomedical Center (RBC), ICAP at Columbia University, and US Centers for Disease Control and Prevention (CDC). Rwanda Population-based HIV Impact Assessment (RPHIA), 2018–2019. https://phia-data.icap.columbia.edu/datasets?country_id=11

11. Ministry of Health, Community Development, Gender, Elderly and Children (MOHCDGEC) [Tanzania], National Bureau of Statistics (NBS), Office of the Chief Government Statistician (OCGS) Zanzibar, ICAP at Columbia University, and US Centers for Disease Control and Prevention (CDC). Tanzania Population-based HIV Impact Assessment (TPHIA), 2016–2017. https://phia-data.icap.columbia.edu/datasets?country_id=10

12. Ministry of Health (MOH) [Uganda], ICAP at Columbia University, and US Centers for Disease Control and Prevention (CDC). Uganda Population-based HIV Impact Assessment (UPHIA), 2016–2017. https://phia-data.icap.columbia.edu/datasets?country_id=7

13. Ministry of Health (MOH) [Zambia], Zambia Statistics Agency (ZamStats), ICAP at Columbia University, and US Centers for Disease Control and Prevention (CDC). Zambia Population-based HIV Impact Assessment (ZAMPHIA), 2016. https://phia-data.icap.columbia.edu/datasets?country_id=5

14. Ministry of Health and Child Care (MOHCC) [Zimbabwe], Zimbabwe National Statistics Agency (ZIMSTAT), ICAP at Columbia University, and US Centers for Disease Control and Prevention (CDC). Zimbabwe Population-based HIV Impact Assessment (ZIMPHIA), 2015–2016. https://phia-data.icap.columbia.edu/datasets?country_id=6

15. Sobel ME. Asymptotic Confidence Intervals for Indirect Effects in Structural Equation Models. *Sociological Methodology*. 1982;13:290-312. doi:10.2307/270723

16. Sobel ME. Some New Results on Indirect Effects and Their Standard Errors in Covariance Structure Models. *Sociological Methodology*. 1986;16:159-186. doi:10.2307/270922

© 2026 He J et al. *JAMA Netw Open*.
