# Supplementary material for: Maternal Engagement in HIV Care and Antiretroviral Therapy Uptake Among Children Living With HIV
Source: JAMA Netw Open. 2026 Aug 26;9(8):e2630983. doi: 10.1001/jamanetworkopen.2026.30983 (PMC13519847; doi:10.1001/jamanetworkopen.2026.30983)
Supplement: Supplementary file 2 [file jamanetwopen-e2630983-s002.pdf]

Data Sharing Statement

He. Maternal Engagement in HIV Care and Antiretroviral Therapy Uptake Among Children Living With HIV. *JAMA Netw Open*. Published August 26, 2026.
doi:10.1001/jamanetworkopen.2026.30983

Data
Data available: No

Additional Information
Explanation for why data not available: The data used in this study were obtained from the Population-based HIV Impact Assessment (PHIA) Project and are the property of the respective national governments and the PHIA Project partners. As this is a secondary analysis of third-party data, the authors are not authorized to share or distribute the datasets. Interested researchers may apply for access to the de-identified datasets through the PHIA through their official data access portal at https://phia-data.icap.columbia.edu/. Access may require a concept note submission and approval from the PHIA Project and relevant national institutional review boards.
